# Comprehensive Registry of Esophageal Cancer in Japan, 2010

**DOI:** 10.1007/s10388-017-0578-4

**Published:** 2017-05-19

**Authors:** Yuji Tachimori, Soji Ozawa, Hodaka Numasaki, Ryu Ishihara, Hisahiro Matsubara, Kei Muro, Tsuneo Oyama, Yasushi Toh, Harushi Udagawa, Takashi Uno, The Registration Committee for Esophageal Cancer of the Japan Esophageal Society

**Affiliations:** 1 Cancer Care Center, Kawasaki Saiwai Hospital, 31-27 Omiya-cho, Saiwai-ku, Kawasaki, Kanagawa 212-0014 Japan; 2 Department of Gastroenterological Surgery, Tokai University School of Medicine, Isehara, Japan; 3 Department of Medical Physics and Engineering, Osaka University Graduate School of Medicine, Osaka, Japan; 4 Department of Gastrointestinal Oncology, Osaka International Cancer Institute, Osaka, Japan; 5 Department of Frontier Surgery, Graduate School of Medicine, Chiba University, Chiba, Japan; 6 Department of Clinical Oncology, Aichi Cancer Center Hospital, Aichi, Japan; 7 Department of Gastroenterology, Saku General Hospital, Nagano, Japan; 8 Department of Gastroenterological Surgery, National Kyushu Cancer Center, Fukuoka, Japan; 9 Department of Gastroenterological Surgery, Toranomon Hospital, Tokyo, Japan; 10 Department of Radiology, Graduate School of Medicine, Chiba University, Chiba, Japan

## Preface 2010

We deeply appreciate the great contributions of many physicians in the registry of esophageal cancer cases. The Comprehensive Registry of Esophageal Cancer in Japan, 2010, was published here, despite some delay. The registry complies with the Act for the Protection of Personal Information. The encryption with a HASH function is used for ‘‘anonymity in an unlinkable fashion’’.

We briefly summarized the Comprehensive Registry of Esophageal Cancer in Japan, 2010. Japanese Classification of Esophageal Cancer 10th and UICC TNM Classification 7th were used for cancer staging according to the subjected year. A total of 5878 cases were registered from 280 institutions in Japan. Tumor locations were cervical: 4.3%, upper thoracic: 12.7%, middle thoracic: 48.8%, lower thoracic: 26.5% and EG junction: 6.5%. Superficial carcinomas (Tis, T1a, T1b) were 34.9%. As for the histologic type of biopsy specimens, squamous cell carcinoma and adenocarcinoma accounted for 90.5 and 4.0%, respectively. Regarding clinical results, the 5-year survival rates of patients treated using endoscopic mucosal resection, concurrent chemoradiotherapy, or esophagectomy were 85.5, 27.3, and 55.5%, respectively. Esophagectomy was performed in 3564 cases. Concerning the approach used for esophagectomy, 30.4% of the cases were treated thoracoscopically. The operative mortality (within 30 days after surgery) was 0.61% and the hospital mortality was 4.29%.

We hope that this Comprehensive Registry of Esophageal Cancer in Japan for 2010 will help to improve all aspects of the diagnosis and treatment of esophageal cancer in Japan.

## Contents

**Fig. 1:**
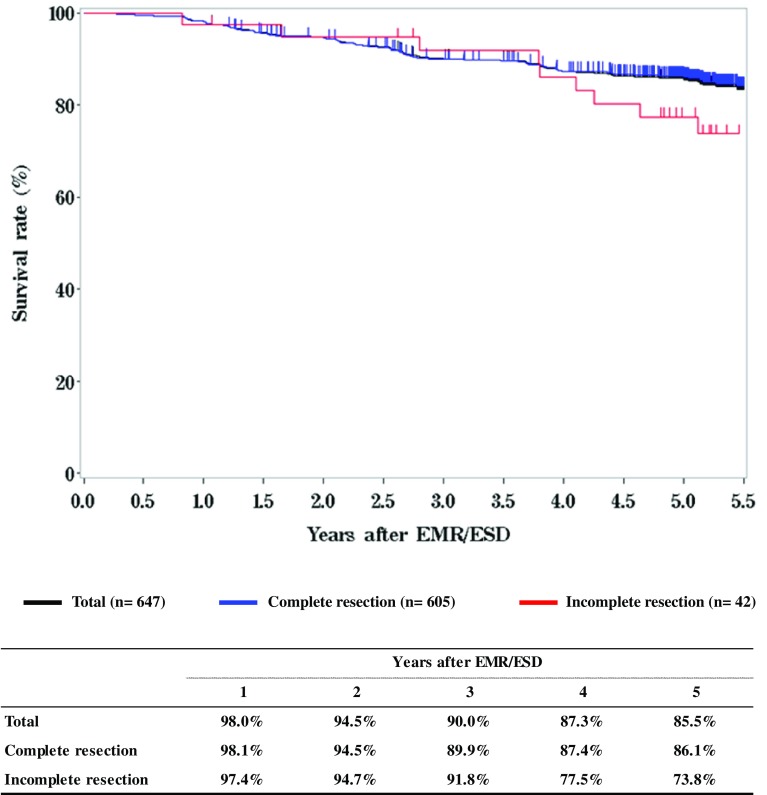
Survival of patients treated with EMR/ESD
**Figure** **2** **Survival of patients treated with EMR/ESD according to the pathological depth of tumor invasion (pT)**

I. **I. Clinical factors of esophageal cancer patients treated in 2010**
  1. **Institution-registered cases in 2010**
  2. **Patient background**
**Table** **1** **Age and gender**
**Table** **2** **Primary treatment**
**Table** **3** **Tumor location**
**Table** **4** **Histologic types of biopsy specimens**
**Table** **5** **Depth of tumor invasion, cT (UICC TNM 7th)**
**Table** **6** **Lymph node metastasis, cN (UICC TNM 7th)**
**Table** **7** **Distant metastasis, cM (UICC TNM 7th)**
**Table** **8** **Clinical Stage (UICC TNM 7th)**
II. **Results of endoscopically treated patients in 2010**
**Table** **9** **Details of endoscopic treatment**
**Table** **10** **Complications of EMR/ESD**
**Table** **11** **Pathological depth of tumor invasion of EMR/ESD specimens**
**Figure** **1** **Survival of patients treated with EMR/ESD**

**Fig. 2:**
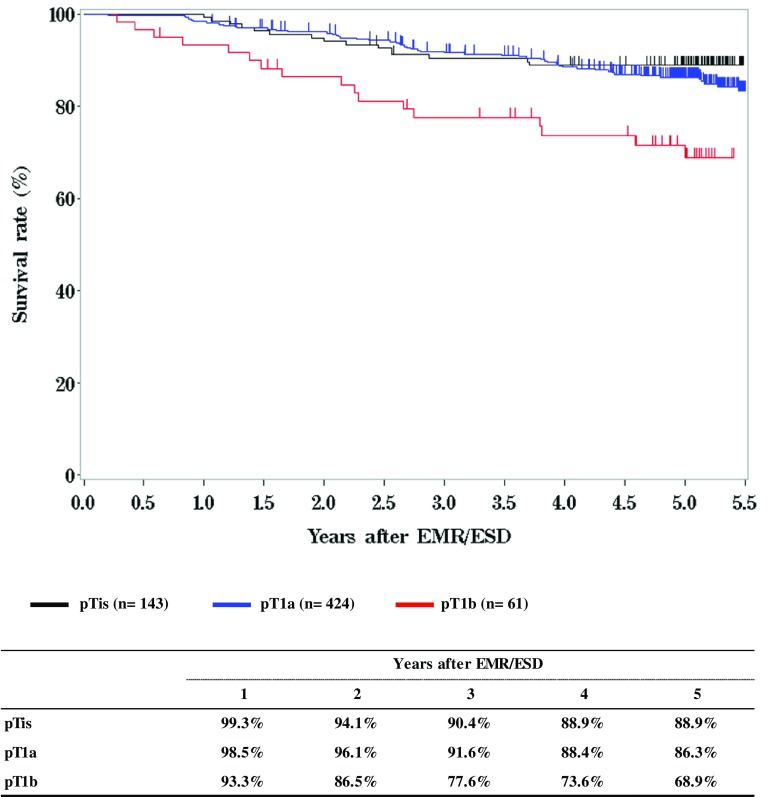
Survival of patients treated with EMR/ESD according to the pathological depth of tumor invasion (pT)
**Figure** **3** **Survival of patients treated with EMR/ESD according to the lymphatic and venous invasion**

**Fig. 3:**
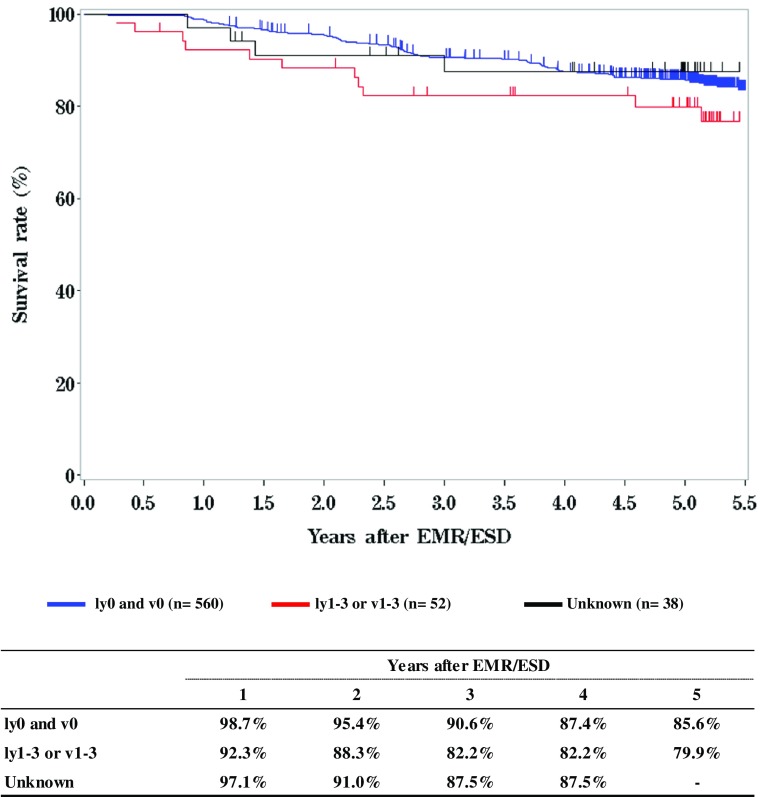
Survival of patients treated with EMR/ESD according to the lymphatic and venous invasion
III. **Results in patients treated with chemotherapy and/or radiotherapy in 2010**
**Table** **12** **Dose of irradiation (non-surgically treated cases)**
**Table** **13** **Dose of irradiation (surgically treated cases)**
**Figure** **4** **Survival of patients treated with chemotherapy and/or radiotherapy**

**Fig. 4:**
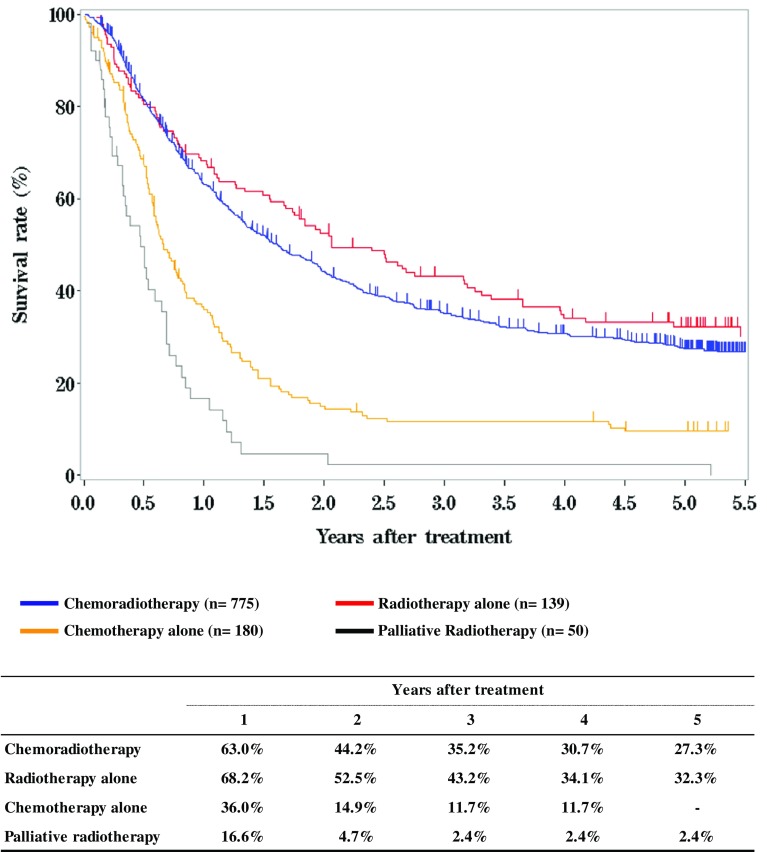
Survival of patients treated with chemotherapy and/or radiotherapy
**Figure** **5** **Survival of patients treated with definitive chemoradiotherapy according to clinical stage (UICC TNM 7th)**

**Fig. 5:**
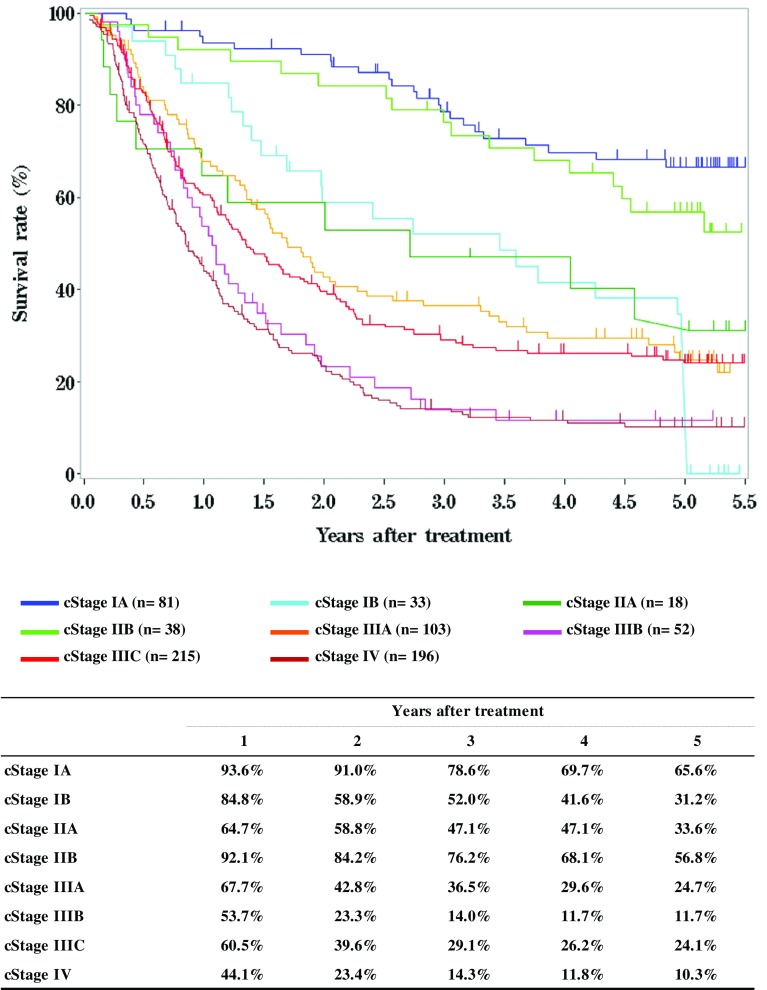
Survival of patients treated with definitive chemoradiotherapy according to clinical stage (UICC TNM 7th)
IV. **Results in patients who underwent esophagectomy in 2010**
**Table** **14** **Treatment modalities of esophagectomy**
**Table** **15** **Tumor location**
**Table** **16** **Approaches to tumor resection**
**Table** **17** **Video-assisted surgery**
**Table** **18** **Fields of lymph node dissection according to the location of the tumor**
**Table** **19** **Reconstruction route**
**Table** **20** **Organs used for reconstruction**
**Table** **21** **Histological classification**
**Table** **22** **Depth of tumor invasion, pT (JES 10th)**
**Table** **23** **Pathological grading of lymph node metastasis, pN (JES 10th)**
**Table** **24** **Pathological findings of lymph node metastasis, pN (UICC 7th)**
**Table** **25** **Pathological findings of distant organ metastasis, pM (JES 10th)**
**Table** **26** **Residual tumor**
**Table** **27** **Causes of death**
**Figure** **6** **Survival of patients who underwent esophagectomy**

**Fig. 6:**
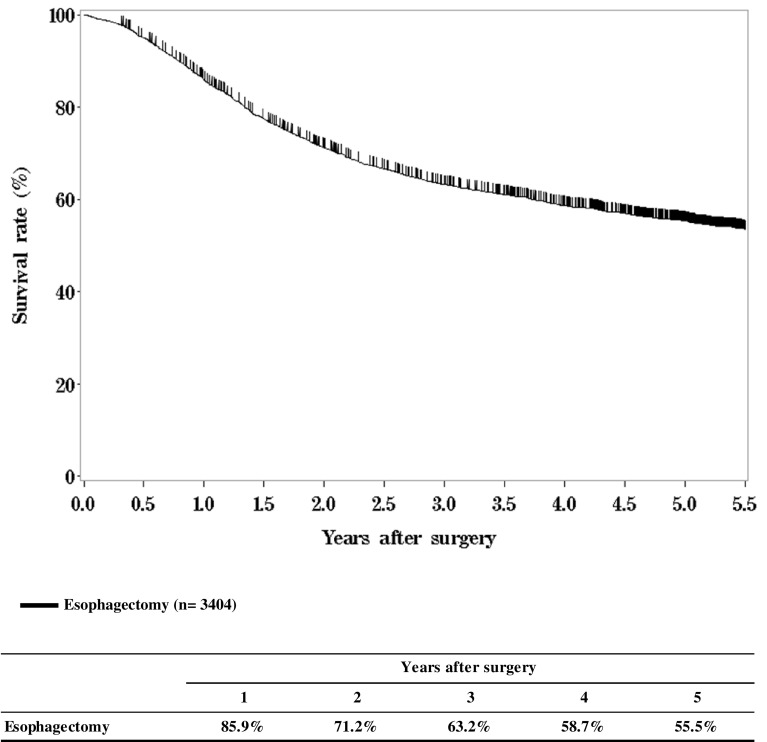
Survival of patients underwent esophagectomy
**Figure** **7** **Survival of patients who underwent esophagectomy according to clinical stage (JES 10th)**

**Fig. 7:**
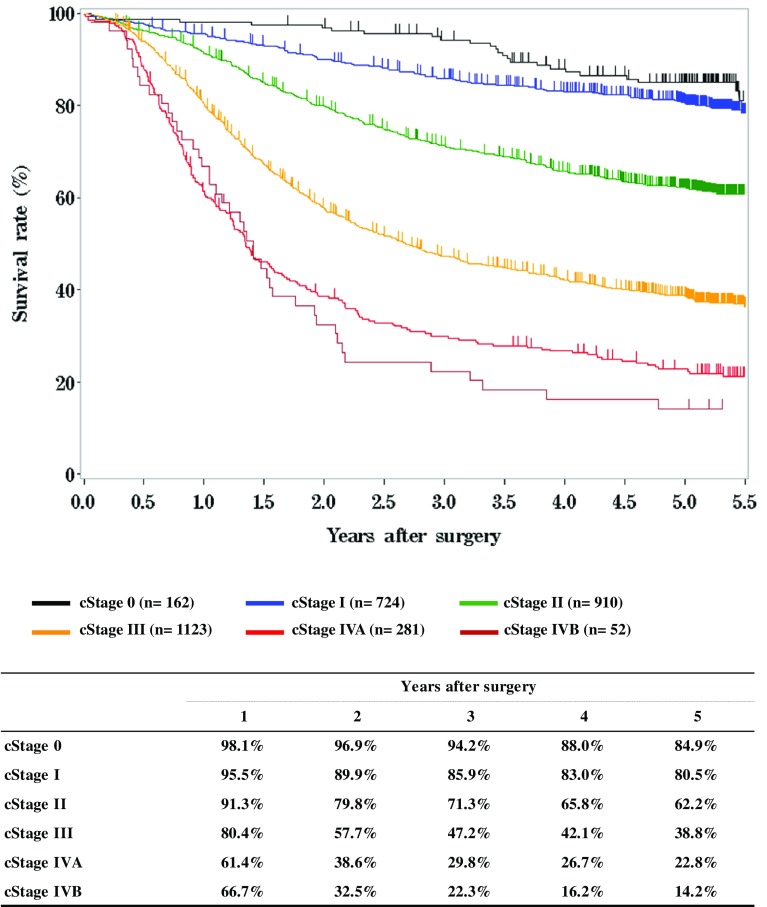
Survival of patients who underwent esophagectomy according to clinical stage (JES 10th)
**Figure** **8** **Survival of patients who underwent esophagectomy according to clinical stage (UICC 7th)**

**Fig. 8:**
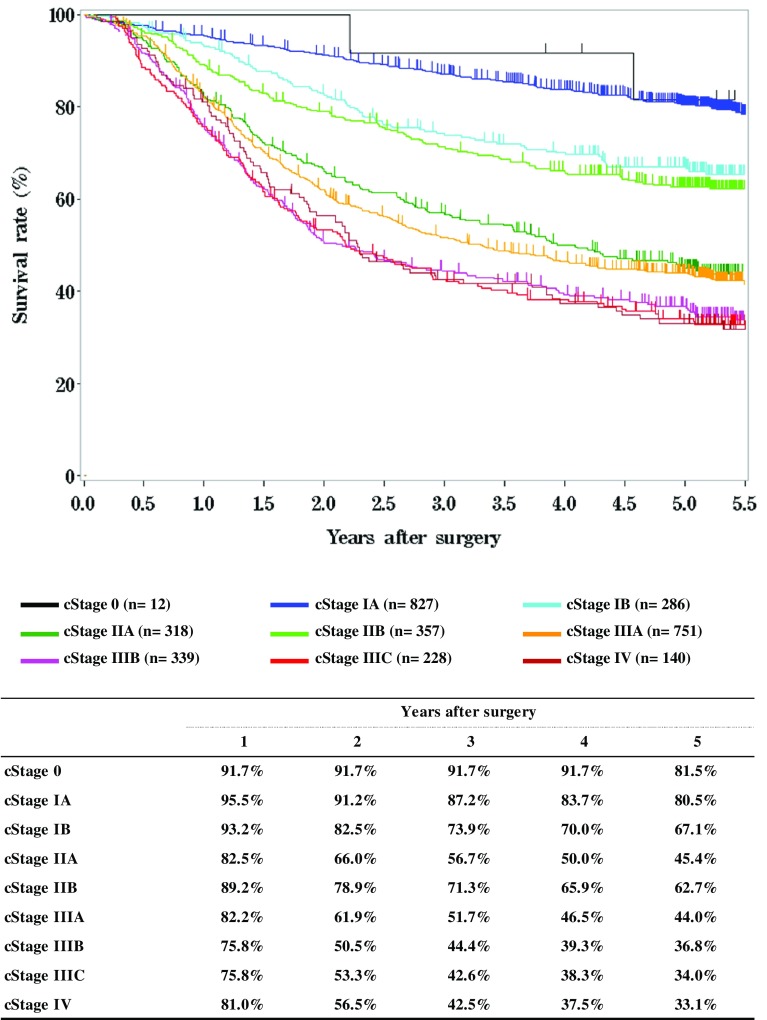
Survival of patients who underwent esophagectomy according to clinical stage (UICC 7th)
**Figure** **9** **Survival of patients who underwent esophagectomy according to the depth of tumor invasion, pT (JES 10th)**

**Fig. 9:**
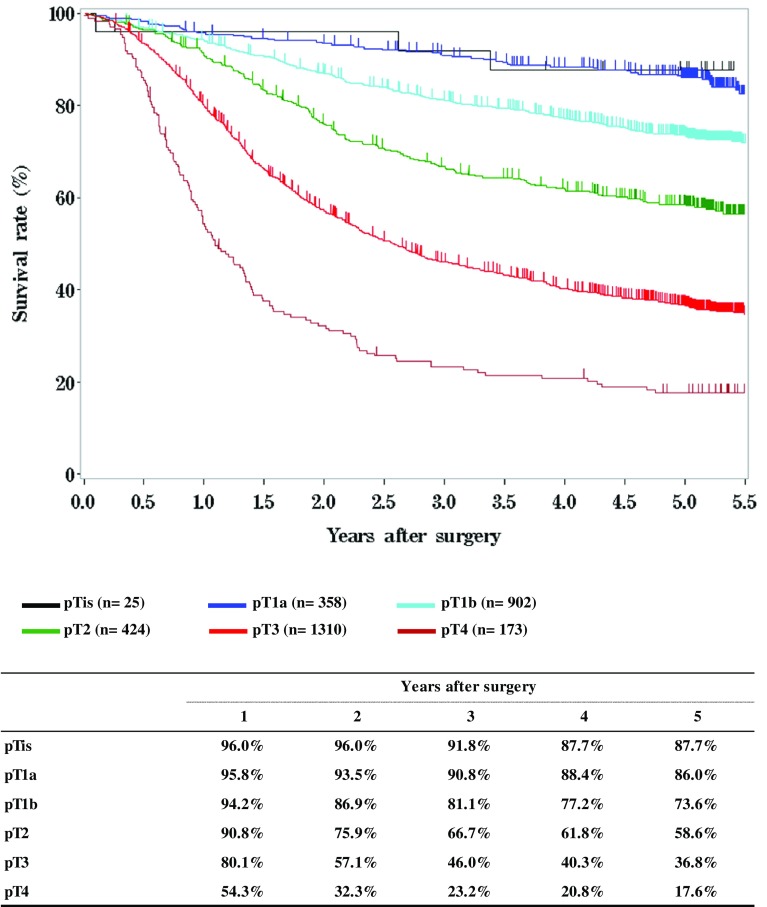
Survival of patients who underwent esophagectomy according to the depth of tumor invasion, pT (JES 10th)
**Figure** **10** **Survival of patients who underwent esophagectomy according to lymph node metastasis, pN (JES 10th)**

**Fig. 10:**
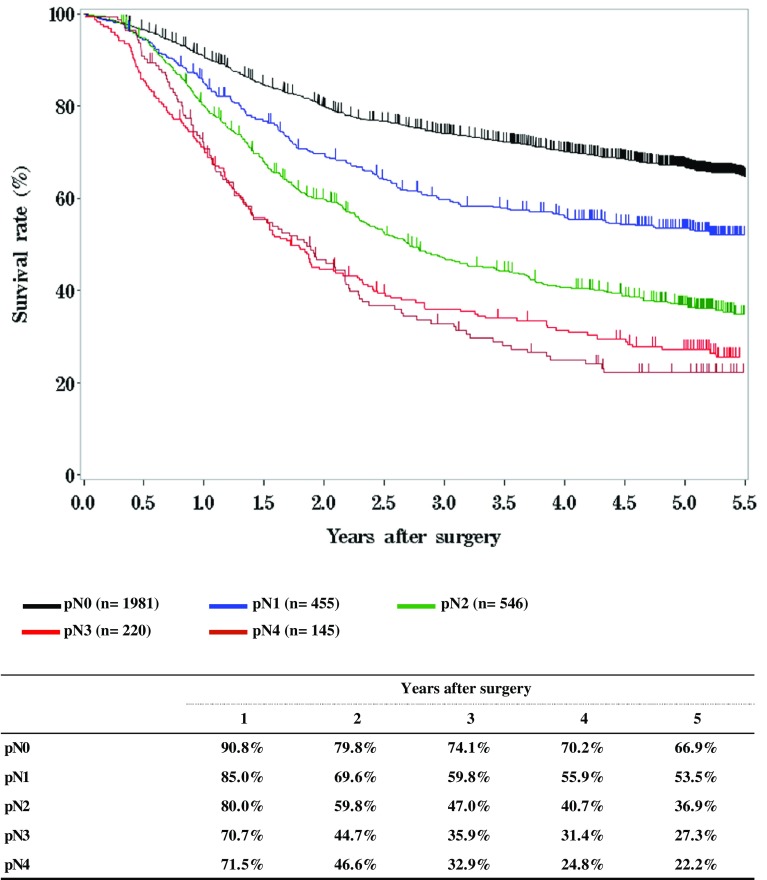
Survival of patients who underwent esophagectomy according to lymph node metastasis, pN (JES 10th)
**Figure** **11** **Survival of patients who underwent esophagectomy according to lymph node metastasis, pN (UICC 7th)**

**Fig. 11:**
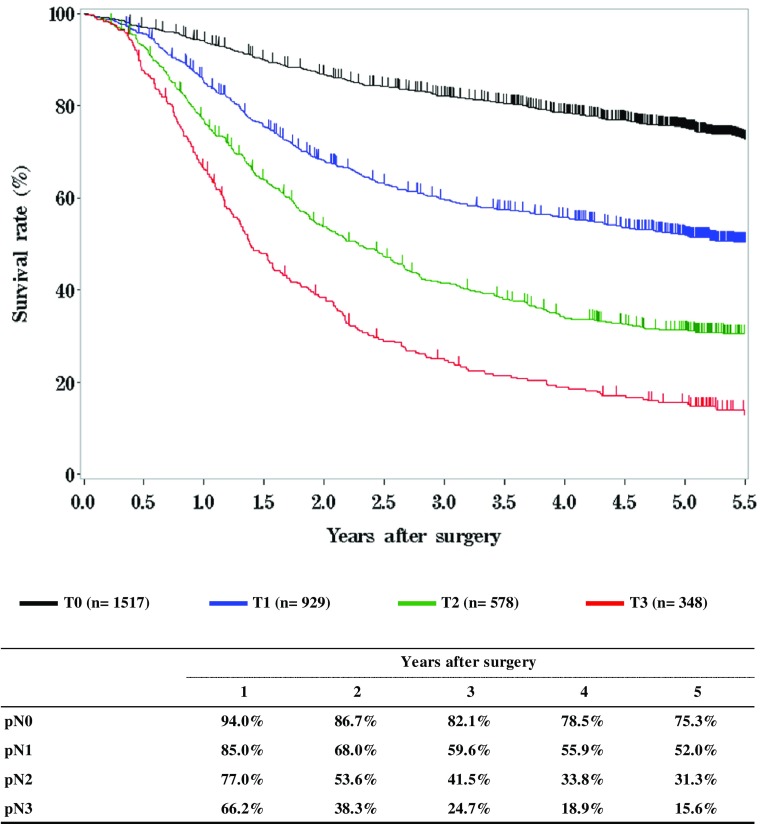
Survival of patients who underwent esophagectomy according to lymph node metastasis, pN (UICC 7th)
**Figure** **12** **Survival of patients who underwent esophagectomy according to pathological stage (JES 10th)**

**Fig. 12:**
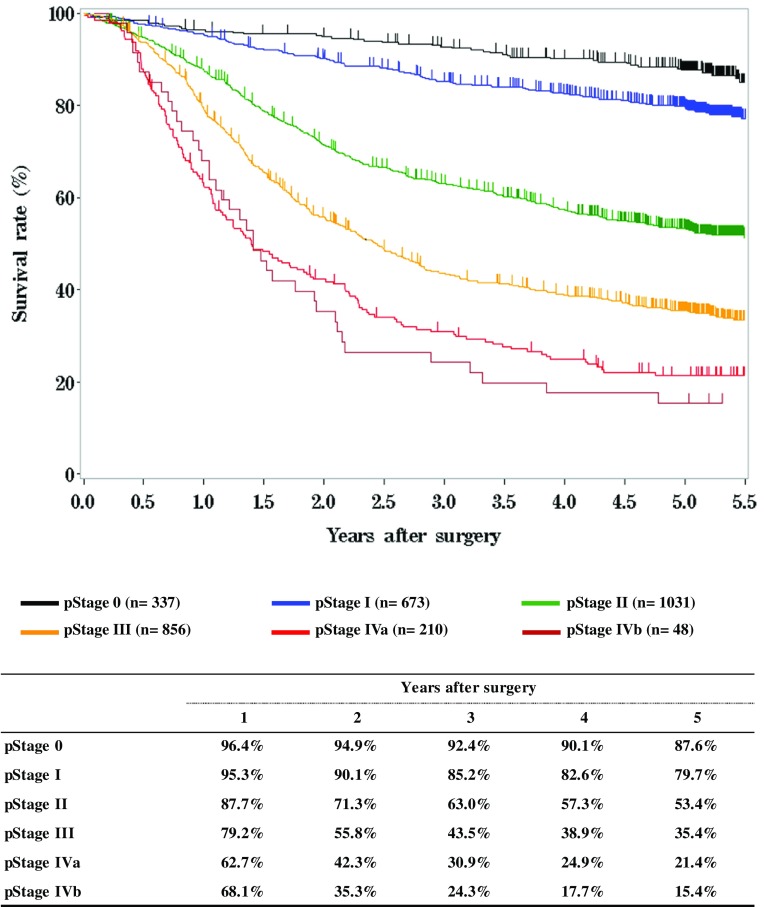
Survival of patients who underwent esophagectomy according to pathological stage (JES 10th)
**Figure** **13** **Survival of patients who underwent esophagectomy according to pathological stage (UICC TNM 7th)**

**Fig. 13:**
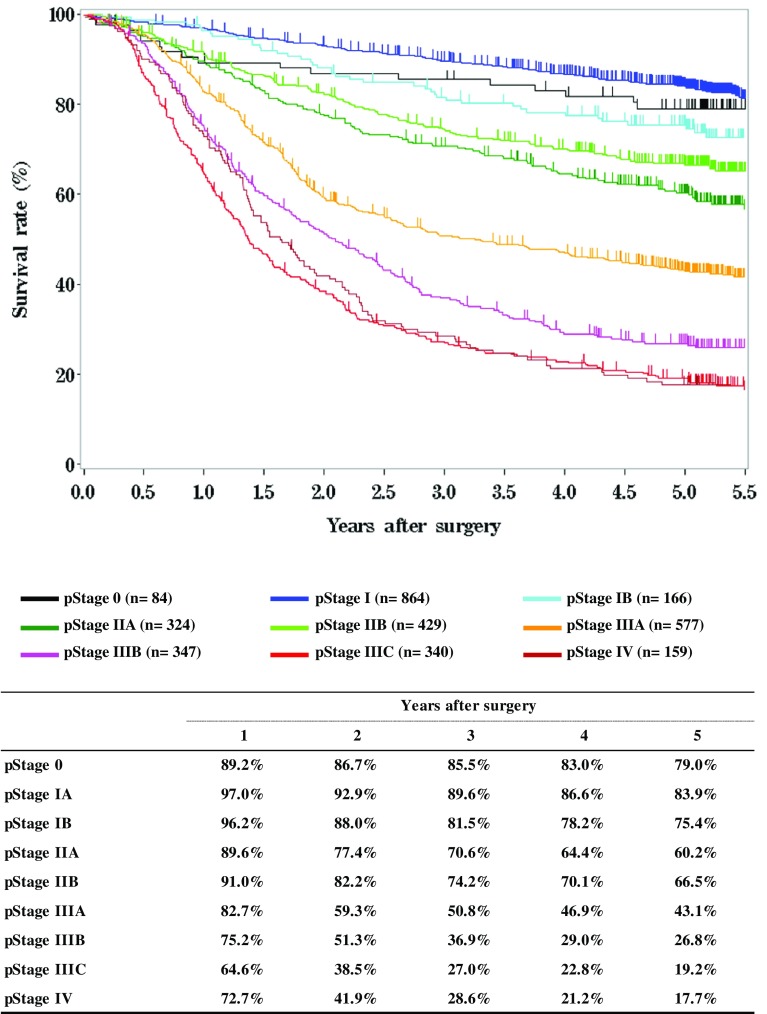
Survival of patients who underwent esophagectomy according to pathological stage (UICC TNM 7th)
**Figure** **14** **Survival of patients who underwent esophagectomy according to residual tumor (R)**

**Fig. 14:**
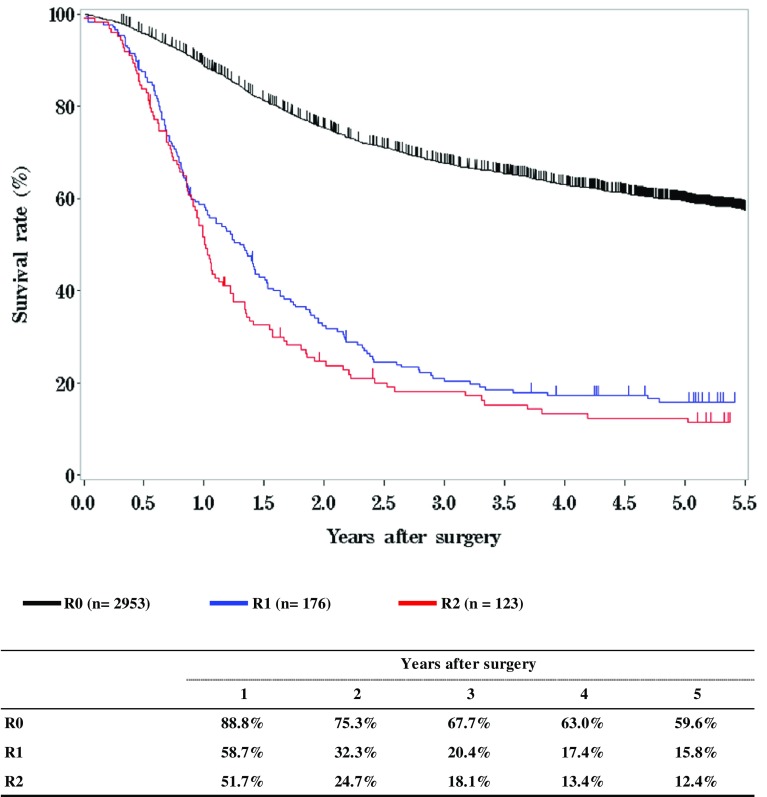
Survival of patients who underwent esophagectomy according to residual tumor (R)

## I. Clinical factors of esophageal cancer patients treated in 2010

### Institution-registered cases in 2010

**Table:** 

Institution
Aichi Cancer Center
Aichi Medical University Hospital
Aizawa Hospital
Akita Kouseiren Hiraka Hospital
Akita University Hospital
Arao Municipal Hospital
Asahikawa Medical College Hospital
Chiba Aoba Municipal Hospital
Chiba Cancer Center
Chiba Medical Center
Chiba Prefectural Sawara Hospital
Chiba University Hospital
Chibaken Saiseikai Narashino Hospital
Dokkyo Medical University Hospital
Ehime University Hospital
Foundation for Detection of Early Gastric Carcinoma
Fuchu Hospital
Fujioka General Hospital
Fujisawa Shounandai Hospital
Fujita Health University
Fukui Prefectural Hospital
Fukui University Hospital
Fukuoka Dental College and Dental Hospital
Fukuoka Saiseikai General Hospital
Fukuoka University Chikushi Hospital
Fukuoka University Hospital
Fukuoka Wajiro Hospital
Fukushima Medical University Hospital
Fukuyama City Hospital
Fussa Hospital
Gifu Prefectural General Medical Center
Gifu University Hospital
Gunma Central General Hospital
Gunma Prefectural Cancer Center
Gunma University Hospital
Gunmaken Saiseikai Maebashi Hospital
Hachinohe City Hospital
Hakodate Goryokaku Hospital
Hakodate National Hospital
Hamamatsu University School of Medicine, University Hospital
Hannan Chuo Hospital
Heartlife Hospital
Higashiosaka City General Hospital
Hiratsuka City Hospital
Hiratsuka Kyosai Hospital
Hirosaki University Hospital
Hiroshima City Asa Hospital
Hiroshima University Research Institute for Radiation Biology Medicine
Hofu Institute of Gastroenterology
Hokkaido Kin-Ikyo Chuo Hospital
Hokkaido University Hospital
Hyogo Cancer Center
Hyogo College of Medicine
Hyogo Prefectural Nishinomiya Hospital
Ibaraki Prefectural Central Hospital
Iizuka Hospital
Ikeda Municipal Hospital
Imazu Surgical Clinic
Inazawa City Hospital
International University of Health and Welfare Hospital
International University of Health and Welfare, Mita Hospital
Isehara Kyodo Hospital
Ishikawa Prefectural Central Hospital
Iwakuni Medical Center
Iwate Medical University Hospital
Iwate Prefectural Chubu Hospital
Japanese Red Cross Fukui Hospital
Japanese Red Cross Ishinomaki Hospital
Japanese Red Cross Kyoto Daini Hospital?
Japanese Red Cross Maebashi Hospital
Japanese Red Cross Nagaoka Hospital
Japanese Red Cross Okayama Hospital
Japanese Red Cross Society Azumino Hospital
Japanese Red Cross Tottori Hospital
Jichi Medical University Hospital
Juntendo University Hospital
Juntendo University Shizuoka Hospital
Junwakai Memorial Hospital
Kagawa Prefectural Central Hospital
Kagawa Rosai Hospital
Kagawa University Hospital
Kagoshima Kenritsu Satsunan Hospital
Kagoshima University Hospital
Kameda General Hospital
Kanagawa Cancer Center
Kanazawa Medical University Hospital
Kanazawa University Hospital
Kansai Medical University Hirakata Hospital
Kansai Medical University Medical Center
Kansai Rosai Hospital
Kasamatsu Hospital
Kashiwa Kousei General Hospital
Kawakita General Hospital
Kawasaki Medical School Hospital
Kawasaki Medical School Kawasaki Hospital
Kawasaki Municipal Ida Hospital
Keio University Hospital
Keiyukai Sapporo Hospital
Kikuna Memorial Hospital
Kinki Central Hospital
Kinki University Hospital
Kiryu Kosei General Hospital
Kishiwada City Hospital
Kitaakita Municipal Hospital
Kitakyushu Municipal Medical Center
Kitano Hospital
Kobe City Medical Center General Hospital
Kobe University Hospital
Kochi Health Science Center
Kochi University Hospital
Kokura Memorial Hospital
Kumamoto City Hospital
Kumamoto University Hospital
Kurashiki Central Hospital
Kurume General Hospital
Kurume University Hospital
Kyoto University Hospital
Kyushu Central Hospital of the Mutual Aid Association of Public School Teachers
Kyushu Hospital
Kyushu Medical Center
Kyushu University Hospital
Machida Municipal Hospital
Matsuda Hospital
Matsushita Memorial Hospital
Matsuyama Red Cross Hospital
Mie University Hospital
Mino City Hospital
Mito Red Cross Hospital
Mitsui Memorial Hospital
Miyazaki Konan Hospital
Murakami General Hospital
Musashino Red Cross Hospital
Nagahama City Hospital
Nagano Red Cross Hospital
Nagaoka Chuo General Hospital
Nagasaki University Hospital
Nagayoshi General Hospital
Nagoya City University Hospital
Nagoya City West Medical Center
Nagoya Daiichi Red Cross Hospital
Nagoya University Hospital
Nanpuh Hospital
Nara Hospital Kinki University Faculty of Medicine
Nara Medical University Hospital
National Cancer Center Hospital
National Cancer Center Hospital East
National Defense Medical College Hospital
National Hospital Organization Beppu Medical Center
National Hospital Organization Chiba-East-Hospital
National Hospital Organization Fukuoka-Higashi Medical Center
National Hospital Organization Iwakuni Medical Center
National Hospital Organization Kure Medical Center
National Hospital Organization Kyoto Medical Center
National Hospital Organization Kyushu Cancer Center
National Hospital Organization Matsumoto National Hospital
National Hospital Organization Nagasaki Medical Center
National Hospital Organization Nagoya Medical Center
National Hospital Organization Osaka National Hospital
National Hospital Organization Tokyo Medical Center
Niigata Cancer Center Hospital
Niigata City General Hospital
Niigata Prefectural Shibata Hospital
Niigata University Medical and Dental Hospital
Nikko Memorial Hospital
Nippon Medical School Chiba Hokusoh Hospital
Nippon Medical School Hospital
Nippon Medical School Musashi Kosugi Hospital
Nippon Medical School Tama Nagayama Hospital
Nishi-Kobe Medical Center
Nishinomiya Municipal Central Hospital
Numazu City Hospital
Obihiro Kousei General Hospital
Obitsusankei Hospital
Ohta General Hospital Foundation Ohta Nishinouchi Hospital
Oita Red Cross Hospital
Oita University Hospital
Okayama Saiseikai General Hospital
Okayama University Hospital
Omuta City Hospital
Osaka City University Hospital
Osaka Hospital of Japan Seafarers Relief Association
Osaka Medical Center for Cancer and Cardiovascular Diseases
Osaka Medical College Hospital
Osaka Police Hospital
Osaka Prefectural Hospital Organization Osaka General Medical Center
Osaka University Hospital
Otsu Municipal Hospital
Otsu Red Cross Hospital
Rinku General Medical Center
Ryukyu University Hospital
Saga-ken Medical Center Koseikan
Saiseikai Fukushima General Hospital
Saiseikai Hiroshima Hospital
Saiseikai Kyoto Hospital
Saiseikai Yahata General Hospital
Saitama Cancer Center
Saitama City Hospital
Saitama Medical Center
Saitama Medical University Hospital
Saitama Medical University Saitama International Medical Center
Saitama Medical University Saitama Medical Center
Sakai City Medical Center
Saku Central Hospital
Sanin Rosai Hospital
Sano Kousei General Hospital
Sendai City Hospital
Sendai Medical Center
Shiga Medical Center for Adults
Shiga University of Medical Science Hospital
Shikoku Cancer Center
Shimada Hospital
Shimane University Hospital
Shimizu Welfare Hospital
Shinshu University Hospital
Shizuoka Cancer Center
Shizuoka City Shizuoka Hospital
Shizuoka General Hospital
Showa University Fujigaoka Hospital
Showa University Hospital
Showa University Koto-Toyosu Hospital
Showa University Northern Yokohama Hospital
Social Insurance Omuta Tenryo Hospital
Social Insurance Tagawa Hospital
St. Marianna University School of Medical Hospital
St. Luke’s International Hospital
Sugita Genpaku Memorial Obama Municipal Hospital
Suita Municipal Hospital
Takasago Municipal Hospital
Takatsuki Red Cross Hospital
Takeda Hospital
Teikyo University Hospital
Teikyo University Hospital, Mizonokuchi
Tenri Hospital
The Cancer Institute Hospital of JFCR
The Jikei University Hospital
The Research Center Hospital for Charged Particle Therapy of NIRS
Tochigi Medical Center
Toho University Omori Medical Center
Toho University Sakura Medical Center
Tohoku Kosai Hospital
Tohoku University Hospital
Tokai University Hospital
Tokushima Municipal Hospital
Tokushima Prefectural Naruto Hospital
Tokushima Red Cross Hospital
Tokushima University Hospital
Tokyo Dental College Ichikawa General Hospital
Tokyo Medical and Dental University Hospital
Tokyo Medical University Hospital
Tokyo Medical University Ibaraki Medical Center
Tokyo Metropolitan Health and Medical Corporation Toshima Hospital
Tokyo University Hospital
Tokyo Women’s Medical University Hospital
Tokyo Women’s Medical University Medical Center East
Tonan Hospital
Toranomon Hospital
Tottori Prefectural Central Hospital
Tottori University Hospital
Toyama Prefectural Central Hospital
Toyama University Hospital
Toyonaka Municipal Hospital
Tsuchiura Kyodo Hospital
Tsukuba University Hospital
Tsuruoka Municipal Shonai Hospital
University Hospital, Kyoto Prefectural University of Medicine
University of Miyazaki Hospital
Wakayama Medical University Hospital
Yamagata Prefectural and Sakata Municipal Hospital Organization
Yamagata Prefectural Central Hospital
Yamagata Prefectural Shinjo Hospital
Yamaguchi University Hospital
Yamaguchi-ken Saiseikai Shimonoseki General Hospital
Yamanashi Prefectural Central Hospital
Yamanashi University Hospital
Yao Municipal Hospital
Yokohama Chuo Hospital
Yokohama City Municipal Hospital
Yokohama City University Hospital
Yokohama City University Medical Center
Yuri General Hospital

(Total 280 institutions)

### **Patient background**

**Table 1:** Age and gender

Age	Male	Female	Unknown	Cases (%)
≤29	1	1		2 (0.0%)
30–39	14	4		18 (0.3%)
40–49	125	44		169 (2.9%)
50–59	802	145	1	948 (16.1%)
60–69	2140	291	1	2432 (41.4%)
70–79	1596	267		1863 (31.7%)
80–89	342	78		420 (7.1%)
≥90	8	7		15 (0.3%)
Unknown	10	1		11 (0.2%)
Total	5038	838	2	5878

**Table 2:** Primary treatment

Treatments	Cases (%)
Surgery	3638 (61.9%)
Esophagectomy	3564 (60.6%)
Palliative	74 (1.3%)
Chemotherapy/radiotherapy	1413 (24.0%)
Endoscopic treatment	827 (14.1%)
Total	5878

**Table 3:** Tumor location

Location of tumor	Endoscopic treatment (%)	Surgery		Chemotherapy and/or radiotherapy (%)	Total (%)
		Esophagectomy (%)	Palliative surgery (%)		
Cervical	18 (2.2%)	116 (3.3%)	6 (8.1%)	114 (8.1%)	254 (4.3%)
Upper thoracic	90 (10.9%)	411 (11.5%)	15 (20.3%)	233 (16.5%)	749 (12.7%)
Middle thoracic	483 (58.4%)	1669 (46.8%)	39 (52.7%)	677 (47.9%)	2868 (48.8%)
Lower thoracic	186 (22.5%)	1054 (29.6%)	12 (16.2%)	303 (21.4%)	1555 (26.5%)
EG	34 (4.1%)	241 (6.8%)	1 (1.4%)	31 (2.2%)	307 (5.2%)
E = G	1 (0.1%)	38 (1.1%)	1 (1.4%)	1 (0.1%)	41 (0.7%)
GE	3 (0.4%)	27 (0.8%)		6 (0.4%)	36 (0.6%)
Unknown	12 (1.5%)	8 (0.2%)		48 (3.4%)	68 (1.2%)
Total	827	3564	74	1413	5878

*E* esophageal, *G* gastric

**Table 4:** Histologic types of biopsy specimens

Histologic types	Cases (%)
Squamous cell carcinoma	5320 (90.5%)
Squamous cell carcinoma	3581 (60.9%)
Well differentiated	334 (5.7%)
Moderately differentiated	1072 (18.2%)
Poorly differentiated	333 (5.7%)
Adenocarcinoma	233 (4.0%)
Barrett’s adenocarcinoma	64 (1.1%)
Adenosquamous carcinoma	8 (0.1%)
Mucoepidermoid carcinoma	2 (0.0%)
Basaloid carcinoma	27 (0.5%)
Endocrine cell carcinoma	13 (0.2%)
Undifferentiated carcinoma	10 (0.2%)
Sarcoma	2 (0.0%)
Malignant melanoma	16 (0.3%)
Carcinosarcoma	9 (0.2%)
GIST	1 (0.0%)
Other tumors	34 (0.6%)
Unknown	139 (2.4%)
Total	5878

**Table 5:** Depth of tumor invasion, cT (UICC TNM 7th)

cT	Cases (%)
cTX	16 (0.3%)
cT0	9 (0.2%)
cTis	144 (2.4%)
cT1a	780 (13.3%)
cT1b	1130 (19.2%)
cT2	813 (13.8%)
cT3	2134 (36.3%)
cT4a	357 (6.1%)
cT4b	410 (7.0%)
Unknown	85 (1.4%)
Total	5878

**Table 6:** Lymph node metastasis, cN (UICC TNM 7th)

cN	Cases (%)
cNX	88 (1.5%)
cN0	2646 (45.0%)
cN1	1642 (27.9%)
cN2	986 (16.8%)
cN3	342 (5.8%)
Unknown	174 (3.0%)
Total	5878

**Table 7:** Distant metastasis, cM (UICC TNM 7th)

cM	Cases (%)
cM0	5091 (86.6%)
cM1	652 (11.1%)
Unknown	135 (2.3%)
Total	5878

**Table 8:** Clinical Stage (UICC TNM 7th)

Clinical stage	Endoscopic treatment (%)	Surgery		Chemotherapy and/or radiotherapy (%)	Total (%)
		Esophagectomy (%)	Palliative surgery (%)		
0	112 (13.5%)	12 (0.3%)		1 (0.1%)	125 (2.1%)
IA	563 (68.1%)	862 (24.2%)		158 (11.2%)	1583 (26.9%)
IB	4 (0.5%)	299 (8.4%)	2 (2.7%)	68 (4.8%)	373 (6.3%)
IIA	2 (0.2%)	336 (9.4%)	7 (9.5%)	49 (3.5%)	394 (6.7%)
IIB	2 (0.2%)	378 (10.6%)	2 (2.7%)	64 (4.5%)	446 (7.6%)
IIIA	7 (0.8%)	783 (22.0%)	12 (16.2%)	165 (11.7%)	967 (16.5%)
IIIB	5 (0.6%)	354 (9.9%)	13 (17.6%)	92 (6.5%)	464 (7.9%)
IIIC	25 (3.0%)	241 (6.8%)	22 (29.7%)	315 (22.3%)	603 (10.3%)
IV	31 (3.7%)	145 (4.1%)	12 (16.2%)	409 (28.9%)	597 (10.2%)
Unknown	76 (9.2%)	154 (4.3%)	4 (5.4%)	92 (6.5%)	326 (5.5%)
Total	827	3564	74	1413	5878

## II. Results of endoscopically treated patients in 2010

**Table 9:** Details of endoscopic treatment

Treatment details	Cases (%)
EMR	59 (7.8%)
EMR + YAG laser	6 (0.8%)
ESD	667 (88.5%)
ESD + EMR	6 (0.8%)
ESD + PDT	4 (0.5%)
ESD + YAG laser	1 (0.1%)
PDT	3 (0.4%)
YAG laser	8 (1.1%)
Total	754

*EMR* endoscopic mucosal resection, *ESD* endoscopic submucosal dissection, *YAG* yttrium aluminum garnet, *PDT* photodynamic therapy

**Table 10:** Complications of EMR/ESD

Complications of EMR/ESD	Cases (%)
None	672 (90.4%)
Perforation	13 (1.7%)
Bleeding	2 (0.3%)
Mediastinitis	4 (0.5%)
Stenosis	44 (5.9%)
Others	8 (1.1%)
Total	743

**Table 11:** Pathological depth of tumor invasion of EMR/ESD specimens

Pathological depth of tumor invasion (pT)	Cases (%)
pTX	1 (0.1%)
pT0	7 (0.9%)
pTis	163 (21.9%)
pT1a	482 (64.9%)
pT1b	74 (10.0%)
pT2	1 (0.1%)
Unknown	15 (2.0%)
Total	743

## III. Results in patients treated with chemotherapy and/or radiotherapy in 2010

**Table 12:** Dose of irradiation (non-surgically treated cases)

Dose of irradiation (Gy)	Definitive		Palliative (%)	Recurrence (%)	Others (%)	Unknown (%)	Total (%)
	Radiation alone (%)	With chemotherapy (%)					
≤29	5 (2.9%)	10 (1.4%)	26 (9.2%)		1 (2.7%)		42 (3.5%)
30–39	1 (0.6%)	7 (1.0%)	43 (15.1%)	3 (10.0%)	4 (10.8%)		58 (4.8%)
40–49	9 (5.3%)	24 (3.5%)	36 (12.7%)	1 (3.3%)	10 (27.0%)		80 (6.6%)
50–59	27 (15.9%)	173 (25.0%)	60 (21.1%)	9 (30.0%)	13 (35.1%)	1 (33.3%)	283 (23.3%)
60–69	124 (72.9%)	453 (65.5%)	109 (38.4%)	17 (56.7%)	9 (24.3%)	2 (66.7%)	714 (58.7%)
≥70	4 (7.2%)	14 (2.1%)	5 (0.0%)				23 (2.2%)
Unknown		11 (1.6%)	5 (1.8%)				16 (1.3%)
Total	170	692	284	30	37	3	1216
Median (min–max)	60.0 (2.0–105.0)	60.0 (1.8–72.0)	52.5 (1.8–90.0)	60.0 (37.5–67.0)	50.0 (9.0–66.0)	60.0 (52.0–60.0)	60.0 (1.8–105.0)

**Table 13:** Dose of irradiation (surgically treated cases)

Dose of irradiation (Gy)	Preoperative irradiation (%)	Postoperative irradiation (%)
≤29	2 (1.0%)	
30–39	34 (17.5%)	1 (2.1%)
40–49	132 (68.0%)	10 (21.3%)
50–59	7 (3.6%)	12 (25.5%)
60–69	13 (6.7%)	22 (46.8%)
≥70		2 (1.1%)
Unknown	6 (3.1%)	
Total	194	47
Median (min–max)	40.0 (0.0–66.0)	60.0 (30.0–79.2)

## IV. Results in patients who underwent esophagectomy in 2010

**Table 14:** Treatment modalities of esophagectomy

Treatments	Cases (%)
Esophagectomy alone	1463 (41.0%)
Esophagectomy + endoscopic treatment	83 (2.3%)
Esophagectomy + chemoradiotherapy	571 (16.0%)
Concurrent chemoradiotherapy	502 (14.1%)
Other	69 (1.9%)
Esophagectomy + chemoradiotherapy + endoscopic treatment	13 (0.4%)
Esophagectomy + chemotherapy	1391 (39.0%)
Preoperative	1027 (28.8%)
Postoperative	183 (5.1%)
Pre and postoperative	75 (2.1%)
Recurrence	46 (1.3%)
Other	60 (1.7%)
Esophagectomy + chemotherapy + endoscopic treatment	3 (0.1%)
Esophagectomy + radiotherapy	39 (1.1%)
Preoperative	4 (0.1%)
Postoperative	7 (0.2%)
Recurrence	15 (0.4%)
Other	13 (0.4%)
Esophagectomy + radiotherapy + endoscopic treatment	1 (0.0%)
Total	3564

**Table 15:** Tumor location

Locations	Cases (%)
Cervical	116 (3.3%)
Upper thoracic	411 (11.5%)
Middle thoracic	1669 (46.8%)
Lower thoracic	1054 (29.6%)
E > G	241 (6.8%)
E = G	38 (1.1%)
G > E	27 (0.8%)
Unknown	8 (0.2%)
Total lesions	3564

**Table 16:** Approaches to tumor resection

Approaches	Cases (%)
Cervical approach	150 (4.2%)
Right thoracotomy	3010 (84.5%)
Left thoracotomy	50 (1.4%)
Left thoracoabdominal approach	58 (1.6%)
Laparotomy	108 (3.0%)
Transhiatal thoracic esophagectomy	50 (1.4%)
Transhiatal lower esophagectomy	72 (2.0%)
Sternotomy	4 (0.1%)
Others	28 (0.8%)
Unknown	34 (1.0%)
Total	3564

**Table 17:** Video-assisted surgery

Video-assisted surgery	Cases (%)
None	2117 (59.4%)
Thoracoscopy	653 (18.3%)
Thoracoscopy + laparoscopy	431 (12.1%)
Thoracoscopy + laparoscopy + mediastinoscopy	1 (0.0%)
Thoracoscopy + laparoscopy + other	1 (0.0%)
Laparoscopy	104 (2.9%)
Laparoscopy + mediastinoscopy	8 (0.2%)
Laparoscopy + other	1 (0.0%)
Mediastinoscopy	4 (0.1%)
Others	11 (0.3%)
Total	3564

**Table 18:** Fields of lymph node dissection according to the location of the tumor

Field of lymphadenectomy	Cervical	Upper thoracic	Middle thoracic	Lower thoracic	E > G	E = G	G > E	Unknown	Total
None	10 (8.6%)	13 (3.2%)	59 (3.5%)	28 (2.7%)	13 (5.4%)			2 (25.0%)	125 (3.5%)
C	36 (31.0%)	10 (2.4%)	20 (1.2%)	3 (0.3%)	1 (0.4%)				70 (2.0%)
C + UM	21 (18.1%)	6 (1.5%)	3 (0.2%)	1 (0.1%)					31 (0.9%)
C + UM + MLM	2 (1.7%)	12 (2.9%)	28 (1.7%)	12 (1.1%)			1 (3.7%)		55 (1.5%)
C + UM + MLM + A	27 (23.3%)	257 (62.5%)	800 (47.9%)	367 (34.8%)	26 (10.8%)	6 (15.8%)		1 (12.5%)	1484 (41.6%)
C + UM + MLM + A+OT				1 (0.1%)					1 (0.0%)
C + UM + A	2 (1.7%)	1 (0.2%)	2 (0.1%)	2 (0.2%)					7 (0.2%)
C + MLM			1 (0.1%)						1 (0.0%)
C + MLM + A	3 (2.6%)	1 (0.2%)	7 (0.4%)	3 (0.3%)					14 (0.4%)
C + A	1 (0.9%)	2 (0.5%)	4 (0.2%)	2 (0.2%)	1 (0.4%)				10 (0.3%)
UM	4 (3.4%)	3 (0.7%)	5 (0.3%)	3 (0.3%)					15 (0.4%)
UM + MLM	1 (0.9%)	7 (1.7%)	29 (1.7%)	12 (1.1%)	1 (0.4%)			1 (12.5%)	51 (1.4%)
UM + MLM + A	3 (2.6%)	75 (18.2%)	627 (37.6%)	478 (45.4%)	56 (23.2%)	5 (13.2%)	1 (3.7%)	1 (12.5%)	1246 (35.0%)
UM + A	1 (0.9%)	4 (1.0%)	2 (0.1%)	2 (0.2%)	2 (0.8%)				11 (0.3%)
MLM		3 (0.7%)	10 (0.6%)	14 (1.3%)	3 (1.2%)				30 (0.8%)
MLM + A	1 (0.9%)	7 (1.7%)	34 (2.0%)	102 (9.7%)	108 (44.8%)	23 (60.5%)	17 (63.0%)		292 (8.2%)
A	1 (0.9%)	6 (1.5%)	22 (1.3%)	12 (1.1%)	28 (11.6%)	3 (7.9%)	8 (29.6%)	1 (12.5%)	81 (2.3%)
Unknown	3 (2.6%)	4 (1.0%)	16 (1.0%)	12 (1.1%)	2 (0.8%)	1 (2.6%)		2 (25.0%)	40 (1.1%)
Total	116	411	1669	1054	241	38	27	8	3564

*C* bilateral cervical nodes, *UM* upper mediastinal nodes, *MLM* middle-lower mediastinal nodes, *A* abdominal nodes

**Table 19:** Reconstruction route

Reconstruction route	Cases (%)
None	57 (1.6%)
Subcutaneous	302 (8.5%)
Retrosternal	1191 (33.4%)
Posterior mediastinal	1473 (41.3%)
Intrathoracic	435 (12.2%)
Cervical	50 (1.4%)
Others	34 (1.0%)
Unknown	22 (0.6%)
Total	3564

**Table 20:** Organs used for reconstruction

Organs used for reconstruction	Cases (%)
None	72 (2.0%)
Whole stomach	71 (2.0%)
Gastric tube	3059 (85.0%)
Jejunum	176 (4.9%)
Free jejunum	69 (1.9%)
Colon	114 (3.2%)
Free colon	10 (0.3%)
Others	13 (0.4%)
Unknown	16 (0.4%)
Total organs	3600
Total cases	3564

**Table 21:** Histological classification

Histological classification	Cases (%)
Squamous cell carcinoma	3045 (86.0%)
Squamous cell carcinoma	584 (16.5%)
Well differentiated	566 (16.0%)
Moderately differentiated	1445 (40.8%)
Poorly differentiated	450 (12.7%)
Adenocarcinoma	161 (4.5%)
Barrett’s adenocarcinoma	71 (2.0%)
Adenosquamous cell carcinoma	20 (0.6%)
Mucoepidermoid carcinoma	3 (0.1%)
Adenoid cystic carcinoma	2 (0.1%)
Basaloid carcinoma	71 (2.0%)
Endocrine cell carcinoma	15 (0.4%)
Undifferentiated carcinoma	6 (0.2%)
Other carcinoma	14 (0.4%)
Carcinosarcoma	18 (0.5%)
Malignant melanoma	16 (0.5%)
GIST	1 (0.0%)
Other	33 (0.9%)
Unknown	66 (1.9%)
Total	3542

**Table 22:** Depth of tumor invasion, pT (JES 10th)

pT category	Cases (%)
pTX	25 (0.7%)
pT0	79 (2.2%)
pTis	25 (0.7%)
pT1a	373 (10.5%)
pT1b	943 (26.5%)
pT2	450 (12.6%)
pT3	1368 (38.4%)
pT4	111 (3.1%)
pT4a	34 (1.0%)
pT4b	33 (0.9%)
Unknown	123 (3.5%)
Total	3564

**Table 23:** Pathological grading of lymph node metastasis, pN (JES 10th)

Lymph node metastasis	Cases (%)
pN0	2079 (58.3%)
pN1	474 (13.3%)
pN2	564 (15.8%)
pN3	233 (6.5%)
pN4	149 (4.2%)
Unknown	65 (1.8%)
Total	3564

**Table 24:** Pathological findings of lymph node metastasis, pN (UICC 7th)

Lymph node metastasis	Cases (%)
pN0	1571 (44.1%)
pN1 (1–2)	956 (26.8%)
pN2 (3–6)	623 (17.5%)
pN3 (7–)	351 (9.8%)
Unknown	63 (1.8%)
Total	3564

Regional lymph nodes are different in JES 10th and UICC 7th

Data for Tables 23 and 24 are analyzed from different variables in the registration application

**Table 25:** Pathological findings of distant organ metastasis, pM (JES 10th)

Distant metastasis	Cases (%)
pMX	62 (1.7%)
pM0	3446 (96.7%)
pM1	56 (1.6%)
Total	3564

**Table 26:** Residual tumor

Residual tumor	Cases (%)
RX	156 (4.1%)
R0	3345 (87.0%)
R1	187 (4.9%)
R2	156 (4.1%)
Total	3844

**Table 27:** Causes of death

Cause of death	Cases (%)
Death due to recurrence	1139 (72.8%)
Death due to other cancer	65 (4.2%)
Death due to other disease (rec+)	44 (2.8%)
Death due to other disease (rec−)	179 (11.4%)
Death due to other disease (rec?)	7 (0.4%)
Operative death*	39 (2.5%)
Postoperative hospital death**	40 (2.6%)
Unknown	52 (3.3%)
Total of death cases	1565 (100%)

*rec* recurrence

* Operative death means death within 30 days after operation in or out of hospital

** Hospital death is defined as death during the same hospitalization, regardless of department at time of death

Operative mortality after esophagectomy: 0.61%

Hospital mortality after esophagectomy: 4.29%

